# Measurement of Zinc Ions in Seawater Samples Using a Microfluidic System Based on the GR/CeO_2_/Nafion Material

**DOI:** 10.3390/molecules29122867

**Published:** 2024-06-16

**Authors:** Wei Tao, Zexi Zeng, Chengjun Qiu, Wei Qu, Yuan Zhuang, Yang Gu, Huili Hao, Zizi Zhao

**Affiliations:** 1 College of Mechanical and Marine Engineering, Beibu Gulf University, Qinzhou 535011, China; 13332493888@163.com (W.T.); zexi1998@163.com (Z.Z.); zhuangyuan@bbgu.edu.cn (Y.Z.); a1059500787@163.com (Y.G.); haohuili0022@163.com (H.H.); sissyzizi@163.com (Z.Z.); 2 Guangxi Key Laboratory of Marine Engineering Equipment and Technology, Qinzhou 535011, China

**Keywords:** electrochemistry, planar disc electrodes, graphene, zinc ions

## Abstract

Considering that heavy-metal contamination of seawater is getting worse, building a quick, accurate and portable device for detecting trace zinc in seawater in real time would be very beneficial. In this work, a microfluidic system was developed that includes a planar disc electrode, a micro-cavity for detection, an electrochemical workstation, a computer, a container for waste liquid reprocessing, an external pipeline and other components as well as a graphene/cerium oxide/nano-cerium oxide/Nafion composite membrane was used to modify the planar disc electrode (GR/CeO_2_/Nafion/Au) to investigate the electrochemical behaviour of Zn(II) using cyclic voltammetry, square-wave voltammetry and orthogonal test methods. Under optimal experimental conditions, the peak reaction current of Zn(II) showed a good linear relationship with the concentration of Zn(II) in the range of 1–900 μg/L with a correlation coefficient of 0.998, and the detection limit of the method was 0.87 μg/L. In addition, the microfluidic system had good stability, reproducibility and anti-interference. The system was used for determining zinc ions in real seawater samples, and the results were very similar to those of inductively coupled plasma–emission spectrometry, demonstrating the practicality of the system for the detection of trace zinc.

## 1. Introduction

Currently, the marine ecosystem is greatly suffering from the excess heavy metal ions produced by domestic and industrial sewage discharges. Abundant zinc amount has a major effect on aquatic life. Zinc is an essential trace element in the sea. According to a previous study, fish, including silver carp and grass carp, cannot grow and develop normally in water containing more than 0.25 mg/L of Zn(II) [1]. According to the World Health Organisation, zinc levels in drinking water should not exceed 3.0 mg/L, and they should be less than 0.1 mg/L according to the Chinese standard for fishery water quality. In descending order of severity, the heavy-metal contamination in the surface water of a given marine area was Pb, Cu, Zn, Hg, Cd and As, with Zn ranking third [2]. In the saltwater and sediments of heavy metal-containing seawater in the sea region of Hailing Island, Guangdong Province, only zinc satisfied the standard for one class of seawater quality, and zinc ranked in the top three in terms of the ability of heavy metals to accumulate in fish [3]. As a result, a novel technique for precise and sensitive detection of zinc ions in marine environments must be developed.

Spectrometry, chromatography, mass spectrometry and electrochemical techniques are now used to detect Zn(II) [4,5,6]. However, the majority of these techniques have a limited range of applications because of their high labour and time requirements, complex pre-treatment procedures and costly equipment. In the field of microfluidics, detection is accomplished through the manipulation of fluids at the micro-metre or even nano-metre scale within a low-dimensional flow channel structure. Its applications span a wide range of industries, including environmental monitoring, biomedicine and the synthesis of useful materials [7]. The use of modern microfluidic systems for environmental pollution detection is becoming more popular because of their high precision, speed and cost-effectiveness [8,9]. At the same time, interest is beginning to grow in the application of these systems for water pollution detection. A more practical and economical approach is produced when electrochemical detection techniques are combined with microfluidics. This is because a microfluidic system requires a small amount of sample, considerably lowering the need for hazardous reagents and making it more economical and environmentally friendly. Additionally, the electrochemical microfluidic technology for zinc ion detection is faster and more portable and provides a higher mass transfer rate as well as a better signal-to-noise ratio than other methods [10].

Graphene (GR) is widely used in metal-ion detection owing to its strong electrocatalytic activity, large specific surface area and low cost [11,12,13,14,15,16]. It is considered to be the ideal material for electrode modification. Moreover, nano-CeO_2_ has been used as an electrode-modification material in electrochemistry because of its remarkable ability to conduct, store oxygen and boost the electron-transfer efficiency in redox processes [17,18,19]. For example, CeO_2_ was used to develop electrochemical sensors that detected trace Zn(II) in a real sample matrix [20].

In this paper, a micro-electromechanical system (MEMS) microfluidic chip and electrochemical principles were used to design and build a microfluidic system, with the planar disc electrode chip serving as its central component. A GR/nano-cerium oxide/Nafion composite membrane was introduced as the zinc ion selective enhancement modification membrane, and the surface of the planar disc electrode was modified using the drop-coating method to endow it with the ability to enrich and separate ions. To achieve fast and extremely sensitive detection of Zn(II) in near-shore seawater based on square-wave voltammetry (SWV), a detection micro-cavity with curved hybrid channels was designed, and a microfluidic system was built in conjunction with the planar disc electrode, an electrochemical workstation and a host computer. Through the use of microfluidic technology, the system compresses the volume of a reactor to the micro-litre scale, considerably reducing specimen volumetric loss while enhancing detection sensitivity. This results in the realisation of an integrated microfluidic system with minimal preparation effort and low detection costs, fulfilling the goal of quickly detecting Zn(II) ions in seawater.

## 2. Materials and Methods

### 2.1. Main Instruments and Reagents

Instruments: an FA224 electronic analytical balance (Shanghai Hailichen Instrument Co., Ltd., Shanghai, China), a TESCAN MIRA LMS high-resolution scanning electron microscope (TESCAN, Brno, Czech Republic), a PLUSE2-5TH ultrapure water system (Nanjing Yipuyida Technology Co., Ltd., Nanjing, China), a JP-040s ultrasonic cleaner (Shenzhen Jiemeng Ultrasonic Instrument Co., Ltd., Shenzhen, China), an LC-LX-H165A high-speed centrifuge (Shanghai Hailichen Instrument Science and Technology Co., Ltd., Shanghai, China), a high-pressure kettle (Shanghai Jie’ang Instrument Co., Ltd., Shanghai, China) and a DHG-101 electric blast drying oven (Shanghai Shangyi Instrument Co., Ltd., Shanghai, China). Electrochemical measurements were performed with a CHI830D electrochemical workstation (Shanghai Chenhua Instrument Co., Ltd., Shanghai, China) using planar disc electrodes (working electrode modified by GR/CeO_2_/Nafion) in a detection micro-cavity.

Reagents: GR (99%; Shanghai Jizi Biochemical Technology Co., Ltd., Shanghai, China), cerium oxide nanoparticles (99.5% purity; Shanghai McLean Biochemical Technology Co., Ltd., Shanghai, China) and a Nafion solution (wt%: 5.0%; Shanghai McLean Biochemical Technology Co., Ltd., Shanghai, China). Other reagents, including glacial acetic acid (CH_3_COOH), anhydrous ethanol (CH_3_CH_2_OH), anhydrous sodium acetate (CH_3_COONa), cadmium nitrate (Cd(NO_3_)_2_), copper nitrate (Cu(NO_3_)_2_) and potassium chloride (KCl) were supplied by Sinopharm Chemical Reagent Beijing Co., Ltd. (Beijing, China) Ultrapure water was used in all experiments; the reagents were all analytically pure.

### 2.2. Design and Construction of Microfluidic System: General Design of the System

**Figure 1:**
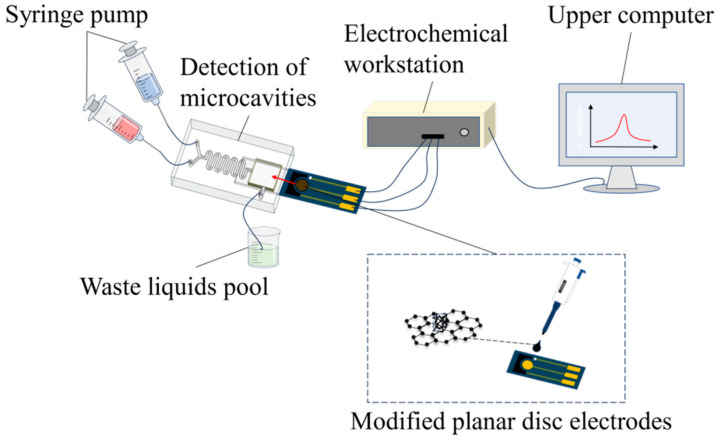
Schematic of the overall structure of the microfluidic system.

A microfluidic chip that could realise the integration and downsizing of multi-unit operation was built according to the structural requirements of the microfluidic chip for operations such as zinc ion detection and the mixing of sample and buffer solutions. Figure 1 depicts the general design plan of the microfluidic chip. The sample inlet and buffer inlet are the two components of the sample inlet. The major components of a microfluidic chip are the mixing area, liquid exit and detection area. First, the corresponding solution was injected into the microfluidic chip by a syringe pump; next, the sample and buffer solutions were thoroughly mixed in the passive mixing zone to create an optimal electrochemical environment for the detection. Subsequently, an electrochemical detector (the modified planar disc electrode) was implanted into the interface of the detection zone. Finally, after the completion of detection, the waste liquid was released into the waste liquid pool via the outlet port. Additionally, wires and adapters were used to link the planar disc electrodes to the electrochemical workstation; USB was used to connect the workstation to the host computer software.

### 2.3. Planar Disc Electrode Design

Based on their forms, electrodes can be categorised as spherical, hemispherical, conical, ribbon-like, circular, disc, array and fork-finger electrodes [21]. To satisfy the requirements of applicability and reliability, this paper uses the disc electrode as the working electrode and integrates a three-electrode system (WE, RE and CE) in the planar disc electrode. The main design is completed for the working electrodes on the planar disc electrodes, and the radius of the working electrodes is determined. This is necessary because the modified electrodes must be implanted into the detection micro-cavity, which requires the largest possible working area.

### 2.4. Detection of Micro-Cavity Mixing Zone Design

Mixing in microfluidic channels can be improved using the following commonly used techniques: changing the internal structure of channels, altering the type of channel at the combination of each channel and altering the fluid flow shape [22]. For improved mixing, we concentrate on the process of altering the internal structure of channels in this study and build a straightforward bending structure.

**Figure 2:**
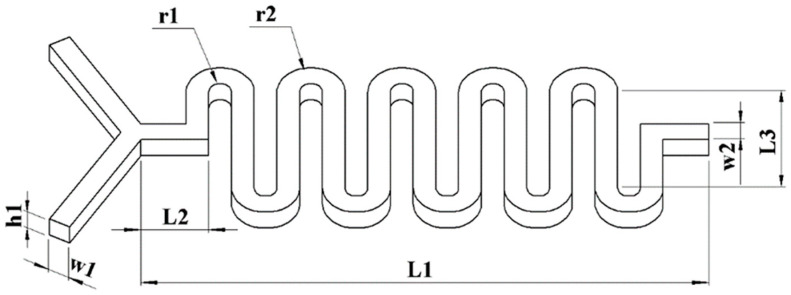
Curved micro-channels.

Based on the idea of increasing mixing efficiency by intensifying sinusoidal fluctuations to encourage fluid collision, the channel used in this study is constructed as a mixing channel with sinusoidal variations (waves). Figure 2 illustrates the following: the mixing distance, L1 = 25 mm; the inlet runner width, w1 = 1 mm; the mixing runner width, w2 = 1 mm; the runner depth, h1 = 1 mm; the inner and outer radii of the bends, r1 = 0.5 mm and r2 = 1.5 mm; the length of the starting mixing straight channel, L2 = 3 mm and the length of the partially DC channel area in the bend, L3 = 6 mm.

### 2.5. Physical Field Simulation Analysis

**Figure 3:**
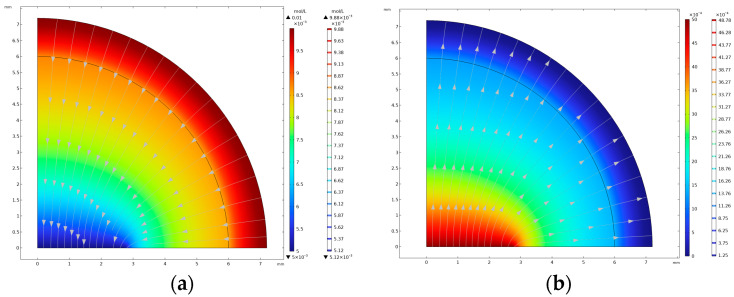
Concentration profiles of (**a**) reductants and (**b**) oxides.

COMSOL 6.0 served as the foundation for the design and simulation of the microfluidic chip structure. Initially, the planar disc electrode structure, the mixing area of the micrometry cavity and the overall structure of the microfluidic chip were constructed. The concentration distribution of oxides and reductants was obtained using modelling analysis, simulation pre-processing and electrochemical field simulation analysis on the planar disc electrode. As shown in Figure 3, the oxides diffuse from the surface of the working electrode to the solution, and the reductants diffuse from the solution to the working electrode, indicating that the working electrode can conduct redox reactions. Then, using the linear scanning results of cyclic voltammetry (CV) and the experimental criteria, the planar disc electrode radius of the ideal structure was found to be 3 mm.

**Figure 4:**
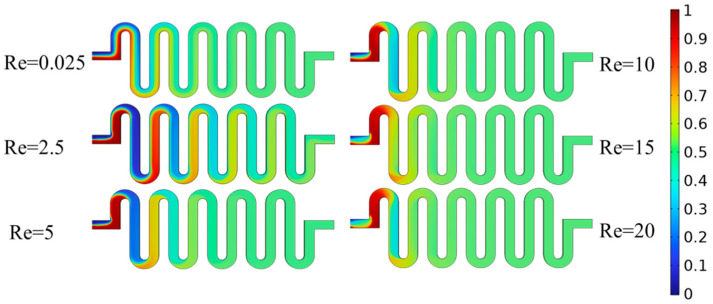
Mixing effects at different Reynolds numbers.

To create an electrolyte solution environment, the sample solution and buffer must be thoroughly combined before zinc ion detection. The design of the detection micro-cavity mixing zone is important because the mixing effect and stability of the two solutions will directly impact the detection results. Adequate mixing not only increases the conductivity of the solution but also removes the influence of electromigration. The detection micro-cavity mixing zone was analysed through modelling, simulation pre-processing and flow field simulation. The sample and buffer substance concentrations were set at 1 and 0 mol/L, respectively. The concentration field distribution was investigated, and the mixing effect was examined under various Reynolds number conditions, as shown in Figure 4, when the Reynolds numbers were 0.025, 2.5, 5, 10, 15 and 20 and when the substance concentration at its exit was ~0.5 mol/L. These results suggest that the mixing effect of this mixing zone is good under various Reynolds numbers.

### 2.6. Construction of the Microfluidic System

**Figure 5:**
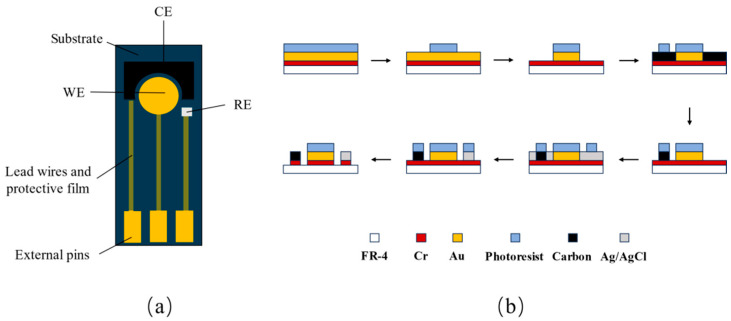
(**a**) Structure of a planar disc electrode and (**b**) fabrication flowchart.

The design strategy specifies that the MEMS technology is used to create the planar disc electrode and the detecting micro-cavity. The material selection for the planar disc electrode is as follows: the FR-4 material was chosen as the planar disc electrode substrate, gold was used as the working electrode, silver/silver chloride was chosen as the reference electrode, and carbon was chosen as the counter electrode. Figure 5a depicts the structure of the planar disc electrode, comprising the substrate, external pins, wires, the working electrode, the counter electrode, the reference electrode and a protective film. The steps involved in preparing planar disc electrodes are depicted in Figure 5b, where the material is sputter deposited, a photoresist is dumped, and the electrode is then etched onto the substrate.

**Figure 6:**
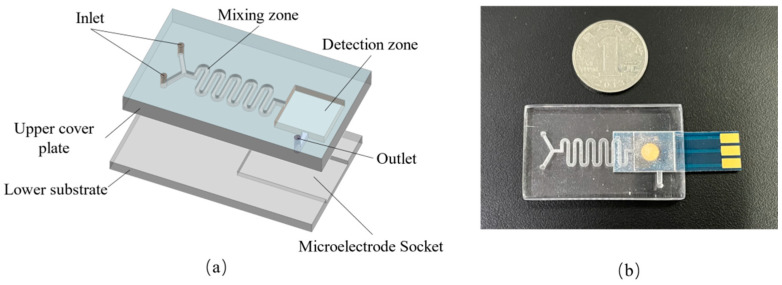
(**a**) Structure and (**b**) physical picture of the microfluidic chip.

The detecting micro-cavity used in this study comprises a lower substrate and an upper cover plate. The planar disc electrode socket is the only component of the lower substrate layer; the upper cover plate contains the inlet port, mixing zone, outlet port and detecting zone. The waste liquid can be discharged to the waste liquid pool through the outlet channel, which has a diameter of 1.5 mm and a length of 4.75 mm. The inlet is a circular channel with a diameter of 1.5 mm. Based on the curved microfluidic mixing zone, a detection zone measuring 12.5 mm × 12.5 mm was designed to be connected with the mixing zone. A planar disc electrode socket measuring 22 mm × 13 mm is located below the detection zone. The detection micro-cavity is made of PDMS, and hot bonding was employed to seal the substrate and upper cover plate. After the cleaning of the lower substrate and the upper cover plate, the substrate surface was treated with a small amount of tetrahydrofuran. Thermal bonding was then performed in a vacuum hot-pressure bonding machine, with a bonding pressure of 15 kgf, a bonding temperature of 120 °C and a bonding duration of 10 min. To create a microfluidic chip, the lower substrate and the upper cover plate were made independently and joined, and their sealing was examined. The components were then assembled using planar disc electrodes. Figure 6b displays the physical picture of the microfluidic chip.

**Figure 7:**
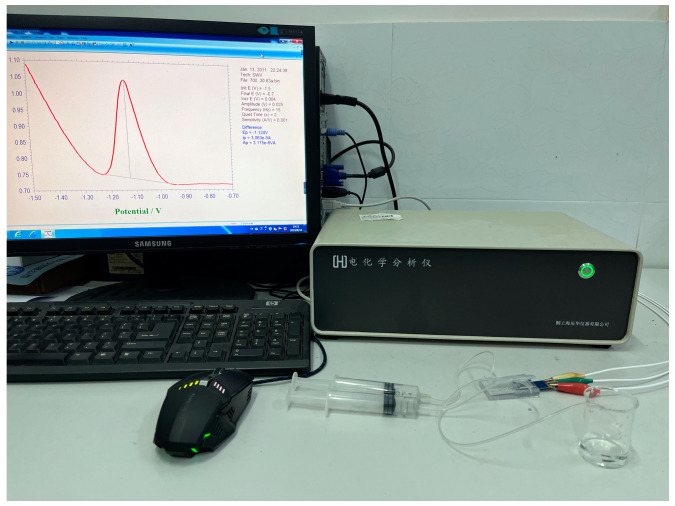
Overall physical diagram of the microfluidic system.

This study examines a system comprising a planar disc electrode, an external connecting conduit, an electrochemical workstation, a computer, a waste liquid recovery tank and a detecting micro-cavity. The polytetrafluoroethylene conduit, which is resistant to heat, acid, alkali and corrosion, was selected for the external connecting conduit used herein in accordance with the experimental requirements. It was sealed with a hot-melt adhesive, and the sealing should be detected after the sealing process was complete. To complete the construction of the entire detection system, the flat disc electrode was inserted into the micro-cavity on the front side, and the external pin of the electrode was connected to the electrochemical workstation, as depicted in Figure 7. Two syringes were used as syringe pumps, and the catheters connected to the two inlet ports were fixed with a solid gel. The catheters at the outlet ports were connected to the waste pool.

### 2.7. Preparation of GR Solution

A homogenous 2.65 mg/mL GR/Nafion solution was obtained by weighing 10.6 mg of GR, dissolving it in 4 mL of anhydrous ethanol solution containing 0.25% Nafion and dispersing it via ultrasonic dispersion for 15 min.

### 2.8. Preparation of the GR/CeO_2_/Nafion-Modified Electrode

To create the GR/Nafion/Au-modified electrode, 5 μL of the GR/Nafion solution was pipetted in a micro-sampler, drop-coated on the surface of a clean planar disc electrode and allowed to dry at room temperature. Then, 2 μL of the 5% Nafion solution was drop-coated on the electrode surface and allowed to dry once more.

To obtain a homogeneous complex solution of 3.0 mg/mL of GR/CeO_2_/Nafion, 0.7 mg of CeO_2_ nanoparticles was added to 2 mL of the GR/Nafion solution and dispersed via ultrasonication for 1 h. Five micro-litres of the GR/CeO_2_/Nafion complex solution were then aspirated into a micro-sampler, drop-coated on the surface of a clean planar disc electrode and allowed to dry at room temperature. To prepare the GR/CeO_2_/Nafion/Au-modified electrode, 2 μL of the 5% Nafion solution was added to the surface of the dried GR/CeO_2_/Nafion-modified electrode and allowed to dry once more.

### 2.9. Electrochemical Detection

With parameters of −1.4 V, a potential increase of 5 mV, a pulse period of 0.2 s, a termination potential of −0.95 V and a pulse width of 0.01 s, Zn(II) was detected via the SWV technique. After adding a specific amount of the Zn(II) standard solution to the HAc–NaAc solution (0.1 mol/L, pH = 5), Zn(II) was added and accumulated using the constant potential method (i–t) for 300 s at a reduction potential of −1.50 V. Applying SWV to oxidise the Zn deposited on the modified film to Zn(II) produced the stripping curve and peaks.

## 3. Results and Discussion

### 3.1. Morphology and Electrochemical Characterisation of Modified Materials

**Figure 8:**
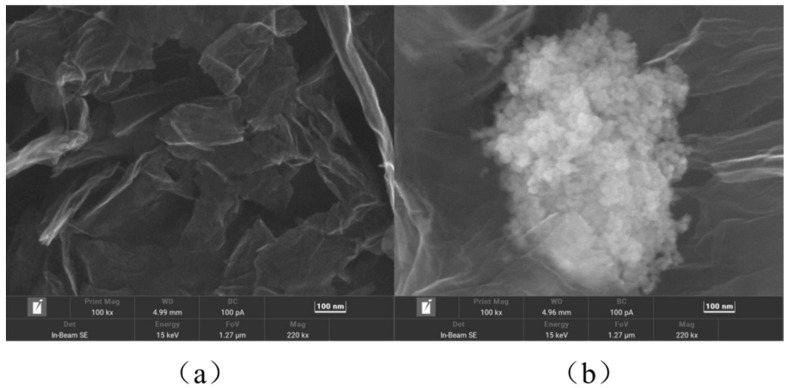
SEM images at a 100 kx magnification for the (**a**) GR material and (**b**) GR/CeO_2_/Nafion composites.

In GR/CeO_2_/Nafion/Au, GR boosts electrical conductivity, CeO_2_ catalyses the reaction and the combination of GR and CeO_2_ gives the material a greater specific surface area and more ion-binding sites. Nafion serves as the binder. Scanning electron microscopy (SEM) was used to characterise the morphology of GR and the GR/CeO_2_/Nafion composite. GR is observed to exhibit a lamellar folded structure with a large surface area, as shown in Figure 8a. The SEM image of the GR/CeO_2_/Nafion composite is displayed in Figure 8b, where the CeO_2_ nano-crystals on the GR lamellar–structure surface can be clearly seen, and CeO_2_ is adhered to the GR surface, suggesting that GR and CeO_2_ have been successfully composited.

**Figure 9:**
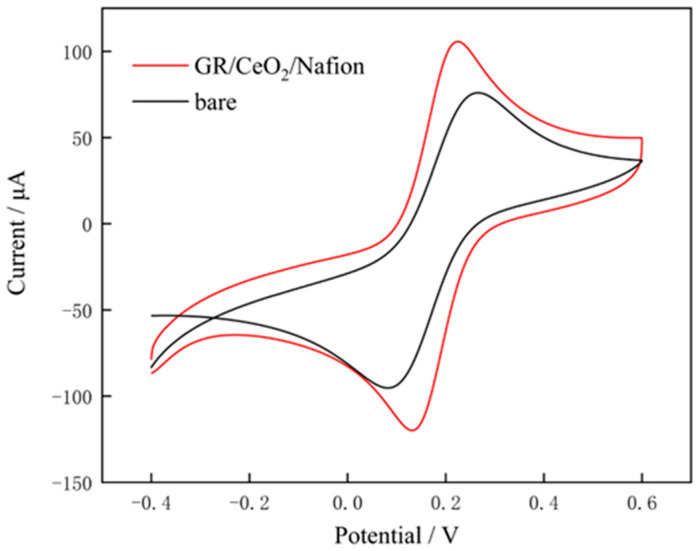
CV curves of bare and GR/CeO_2_/Nafion-modified electrodes in a 0.1 mol/L potassium ferricyanide solution.

The CV curves in a 0.1 mol/L potassium ferricyanide solution for the bare electrode and the GR/CeO_2_/Nafion-modified electrode are displayed in Figure 9. Their comparison demonstrates that the peak redox current of the bare electrode is lower than that of the GR/CeO_2_/Nafion-modified electrode. The following equations may be employed to determine the outcome:

$$∆E_{P}=E_{Pa}-E_{Pc}$$

where $∆E_{P}$, $E_{Pa}$ and $E_{Pc}$ represent the potential difference, oxidation peak potential and reduction peak potential, respectively. The reasons for this observation are as follows: On the one hand, CeO_2_ nanoparticles on the surface of the modified electrode form a ‘fast electron ladder’ that considerably accelerates electron transfer, and Ce ions are easily converted from trivalent to tetravalent, resulting in a large number of active sites in the reaction; on the other hand, the large surface area of GR allows it to adsorb a large number of CeO_2_ nanoparticles. The active sites on the surface of CeO_2_ nanoparticles and the active sites on GR that are not occupied by CeO_2_ nanoparticles allow the analytes to be adsorbed, and they work together to speed up the electron transfer.

### 3.2. Detection Performance of Modified Electrodes

**Figure 10:**
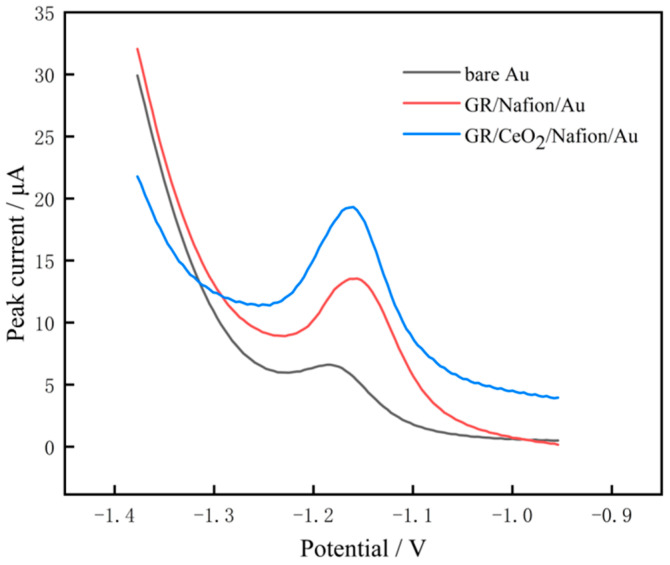
SWV plots of different electrodes in a buffer solution containing 400 μg/L of Zn(II).

The SWV approach was used to compare the signal responses of three electrodes to Zn(II) (400 μg/L): the bare, GR/Nafion-modified and GR/CeO_2_/Nafion-modified electrodes. The Zn(II) dissolution peak potential, represented by −1.1 V in Figure 10, was better shaped and had a larger peak current for all three electrodes. However, the GR/CeO_2_/Nafion/Au-modified electrode displayed the strongest Zn(II) response signal because, after the addition of the composite of GR and CeO_2_ nanoparticles, the electrode surface exhibited an increased number of active sites, an increased electron mobility rate and an enhanced dissolution peak current. This is because the combination of GR and CeO_2_ nanoparticles increases the number of active sites on the electrode surface, accelerates the flow of electrons and amplifies the dissolution peak current.

### 3.3. Optimisation of Electrochemical Detection Conditions

#### 3.3.1. Optimisation of Buffer Solution pH

**Figure 11:**
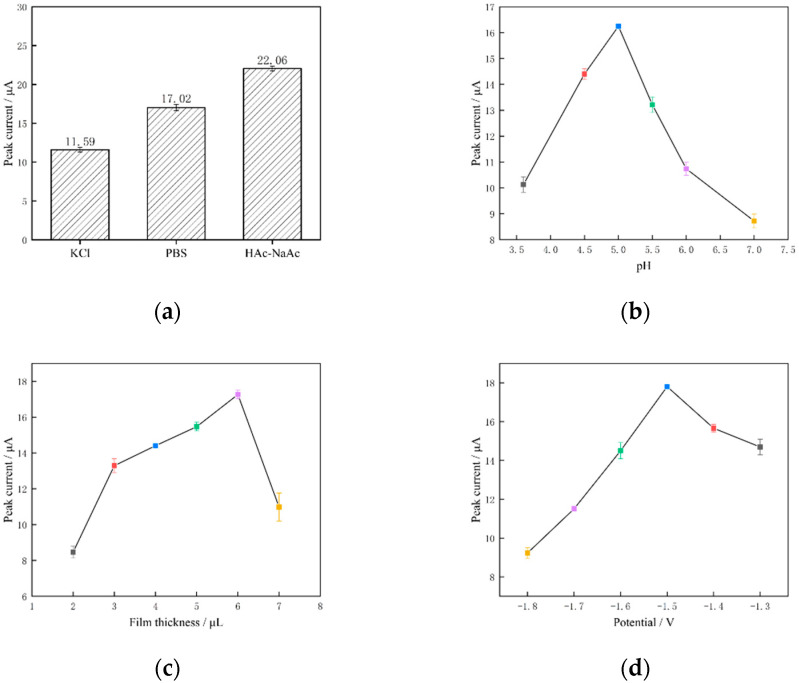

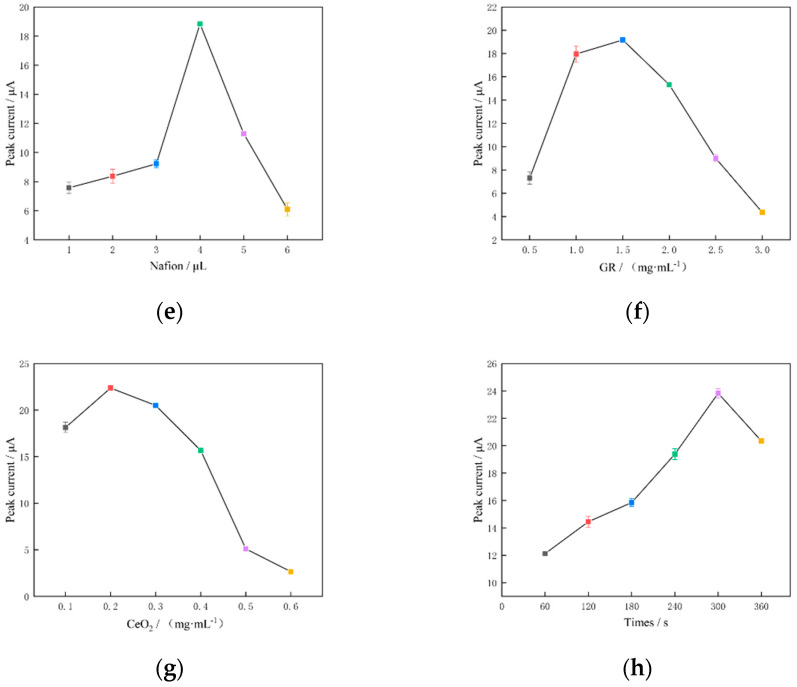
(**a**) Buffer type, (**b**) pH, (**c**) membrane thickness, (**d**) enrichment potential, (**e**) Nafion modification amount, (**f**) GR concentration, (**g**) CeO_2_ concentration and (**h**) enrichment time.

SWV was performed in a potassium chloride buffer solution (KCl), a phosphate buffer solution and an acetate buffer solution (HAc–NaAc), all of which contained 600 μg/mL of zinc ions, and the concentrations of all three buffer solutions were 0.1 mol/L, and their pH values were 6.0. The SWV measurement results are shown in Figure 11a. The peak current value of the zinc ions measured in the HAc–NaAc buffer solution was 22.06 μA, which was greater than that of the remaining two buffer solutions. The current peaked at 22.06 μA, which was larger than the remaining two buffer solutions, so the HAc–NaAc solution was chosen as the buffer solution in this study.

The influence of pH on the Zn(II) dissolution voltammetric response was obtained by examining the corresponding Zn(II) current response peaks in Figure 11b and increasing the pH of the 0.1 mol/L HAc–NaAc buffer solution from 3.6 to 7.0. Specifically, when the pH of the HAc–NaAc buffer solution was increased from 3.6 to 5.0, the peak current response gradually increased and reached its maximum value at pH = 5.0. Conversely, when the pH was raised from 5.0 to 7.0, the peak current response progressively decreased. A pH level that is too high will cause zinc ions to hydrolyse and produce metal hydroxides and complexes, which will lower the concentration of zinc ions in the solution. A pH value that is too low will intensify the hydrogen precipitation reaction and impede the deposition process. Therefore, 5.0 is the ideal pH value that was chosen for this study.

#### 3.3.2. Optimisation of Film Thickness

Variation in zinc ion peak current response with varying film thicknesses was studied in the 2–7 μL range; Figure 11c displays the measured values. The peak current responsiveness of the film increased as the film thickness increased within the range of 2–6 μL. The highest peak current response value of the GR/CeO_2_/Nafion material to zinc ions was attained at a film thickness of 6 μL. As the film thickness increased, the peak value of the current response of the dissolving peak decreased. This problem is mostly caused by an excessively thick coating, which is detrimental to electron transfer on the electrode surface. Therefore, a film thickness of 6 μL was selected in this study.

#### 3.3.3. Optimisation of Enrichment Potentials

Zinc ion peak current response was examined in relation to enrichment potentials between −1.8 and −1.3 V. The measured results are displayed in Figure 11d. The peak current response increased over time when the deposition potential was increased from −1.8 to −1.5 V. The peak current response was the maximum at a deposition voltage of −1.5 V, and the current response peak of the electrode tended to decrease. Zinc ion precipitation and desorption were impacted by a strong hydrogen precipitation reaction on the electrode surface at high potentials. However, complete reduction of zinc ions was impossible at higher potentials than −1.5 V, which is why −1.5 V was selected as the enrichment potential for the entire experiment.

#### 3.3.4. Optimisation of the Nafion Modification Amount

As Nafion is a cation-exchange membrane, adding it to the composite solution may have binding effects. The anti-disturbance effect and the exchange features of Zn(II) detection are strengthened when the Nafion membrane is coated on the electrode, in addition to increasing conductivity. Figure 11e displays the findings of the investigation on the impact of Nafion modification amount on Zn(II) peak current response in the range of 1–6 μL. The current response peak increased gradually from 1 to 4 μL, reaching its maximum value at 4 μL; conversely, when the modification amount was increased from 4 to 6 μL, the current response peak progressively decreased. The explanation for this is that a thick Nafion membrane may impede the redox reaction and alter zinc ion exchange. It may also cause the modification material to separate, reducing the effectiveness of detection. Therefore, in this study, 4 μL was selected as the optimal amount for Nafion modification.

#### 3.3.5. Optimisation of the GR Concentration

The primary factor influencing the detection of zinc ions is the concentration of GR; the concentration range of 0.5–3.0 mg/mL was investigated to determine the effect of GR concentrations on the peak value of the current response. As the GR concentration was increased from 0.5 to 1.5 mg/mL, as shown in Figure 11f, the current peak gradually increased, reaching its maximum value at 1.5 mg/mL. Conversely, as the GR concentration was increased from 5.5 to 3.0 mg/mL, the current peak gradually decreased. The adsorption capacity of zinc ions on the electrode surface is influenced by the GR concentration; however, excessive GR concentrations cause high background currents that obstruct zinc ion detection. Therefore, the GR concentration was selected as 1.5 mg/mL in this study.

#### 3.3.6. Optimisation of the CeO_2_ Concentration

Zinc ion detection is significantly impacted by the CeO_2_ concentration. The impact of CeO_2_ concentrations on the current response peak was studied within the 0.1–0.6 mg/mL range. As shown in Figure 11g, when the concentration of CeO_2_ was increased from 0.1 to 0.2 mg/mL, the current response peak reached its maximum value (at 0.2 mg/mL). The current peak then gradually decreased as the concentration of CeO_2_ was increased from 0.2 to 0.6 mg/mL. This is explained by the fact that CeO_2_ will enhance the number of electrode surface active sites, zinc ion adsorption capacity and electron-transfer rate during the redox reaction. However, the concentration of CeO_2_ is too high to impact the electrode’s electrical conductivity or obstruct the detection of zinc ions. Therefore, the CeO_2_ concentration in this paper was selected as 0.2 mg/mL.

#### 3.3.7. Optimisation of Enrichment Time

The measured outcomes of the investigation on the association between the peak Zn(II) current response and the enrichment time are displayed in Figure 11h. The peak current response increased with increasing enrichment time in the range of 0–300 s. When the enrichment time increased beyond 300 s, the peak current response decreased. This phenomenon is primarily caused by the long-term enrichment saturating the active sites on the electrode surface, leading to a hydrogen precipitation reaction during the enrichment process and the subsequent fall-off of the enriched zinc from the electrode surface. The enrichment time of 300 s was selected for this study based on the peak condition of the current response and the effectiveness of the experimental analysis.

#### 3.3.8. Orthogonal Experiments

Orthogonal experiments were conducted to verify and determine whether the obtained composite films were optimal. Only the three distinct concentrations of each component needed to be considered because only three components were used in this investigation to manufacture zinc ion selectively enhanced films. This allowed the optimum conditions to be verified in the smallest number of experiments. The optimum concentration of each and its two closest to the optimum concentrations were chosen in this case, and a table of orthogonal test protocols was established. Experiments were conducted, and the results are displayed in Table 1.

To establish the ideal level of each component, the mean and extreme deviation of the orthogonal test findings must be analysed. To obtain the orthogonal test intuitive analysis of the table (Table 2), nine experiments must be conducted three times at each level. Then, the mean and extreme deviations of the outcomes of the three experiments should be computed at the same level.

The disparities between the three levels can be reflected in the magnitude of the experimental mean value, and the larger the extreme difference, the more the impact of the various levels of the experimental results. The orthogonal test visual analysis presented in Table 2 shows that the highest peak of the zinc ion dissolution response current at Level 2 is shared by all four components. A GR concentration of 1.5 mg/mL, a CeO_2_ concentration of 0.2 mg/mL, a Nafion modification amount of 4 μL and a film thickness of 6 μL were found to be the best levels. The experimental settings established in this study are accurate and ideal, as proven by the results of orthogonal tests, which agree with the outcomes of the optimised experimental conditions in the prior paper.

### 3.4. Detection of Zinc Ions

**Figure 12:**
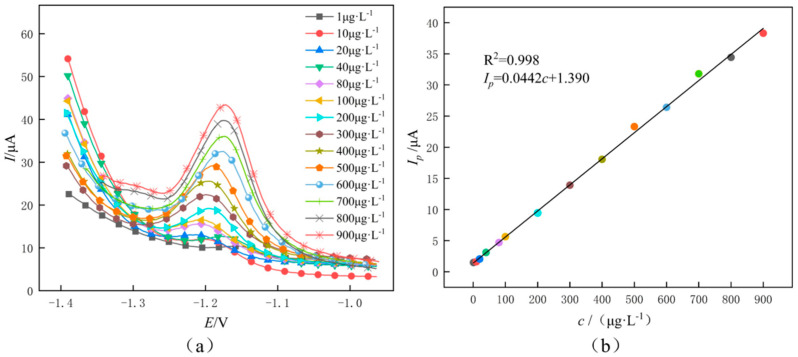
(**a**) Zn dissolution voltammetric response curves of GR/CeO_2_/Nafion-modified planar disc electrodes for zinc ions at different concentrations (1−900 μg/L). (**b**) Linear regression curves with their corresponding.

Zinc ions were detected using a MEMS microfluidic system, and at various Zn(II) concentrations, the detection performance of GR/CeO_2_/Nafion/Au was assessed using a standard curve of zinc ion concentration established via SWV detection under ideal conditions. The Zn(II) response currents of GR/CeO_2_/Nafion/Au in the concentration range of 1–900 μg/L are displayed in Figure 12. The SWV response peak current in this range is linearly correlated with an *R*^2^ coefficient of 0.998; the relative standard deviation (RSD) of Zn(II) is 1.28%, and the linear regression equation is *ip* = 0.0442 c + 1.390 (where *ip* is the dissolution peak current and *c* is the concentration of Zn(II)). The Zn(II) detection limit of the system is 0.87 μg/L (calculated by 3*σ*/m, where *σ* is the RSD of Zn(II) with a 1 μg/L concentration and *m* is the slope of the linear equation). This is less than the limits for Zn(II) content in drinking water allowed by WHO and Chinese standards.

### 3.5. Stability, Reproducibility and Anti-Interference Experiments of Modified Electrodes

**Figure 13:**
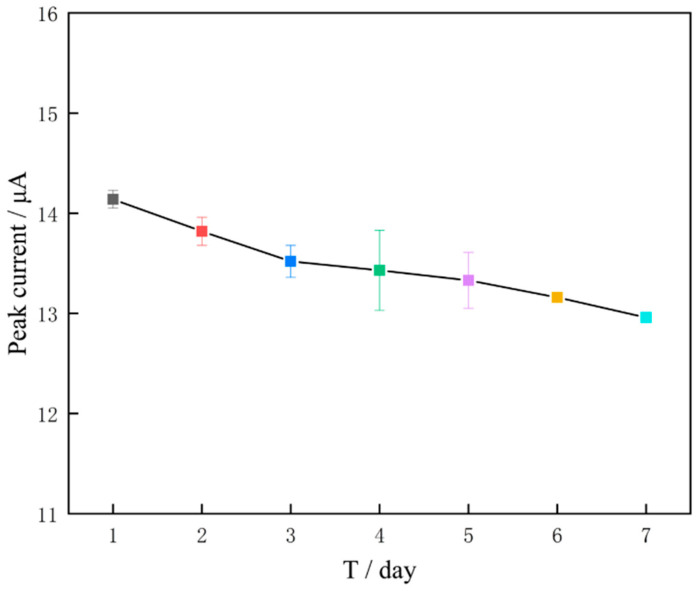
Trend of peak current change for seven consecutive days.

To assess the stability of GR/CeO_2_/Nafion/Au, the prepared material was refrigerated at 4 °C for a week to determine 300 μg/L of Zn(II). The peak current of the electrode changed by no more than 10% after every 24 h of storage, indicating that the modified electrode was stable (Figure 13).

Five batches of planar disc electrodes were modified using the same GR/CeO_2_/Nafion composite membrane to create five GR/CeO_2_/Nafion/Au electrodes. These were then measured in a buffer solution containing 300 μg/L of Zn(II), and the RSD of the dissolved peak current was 3.5%, indicating good reproducibility of the electrode-modification process.

**Figure 14:**
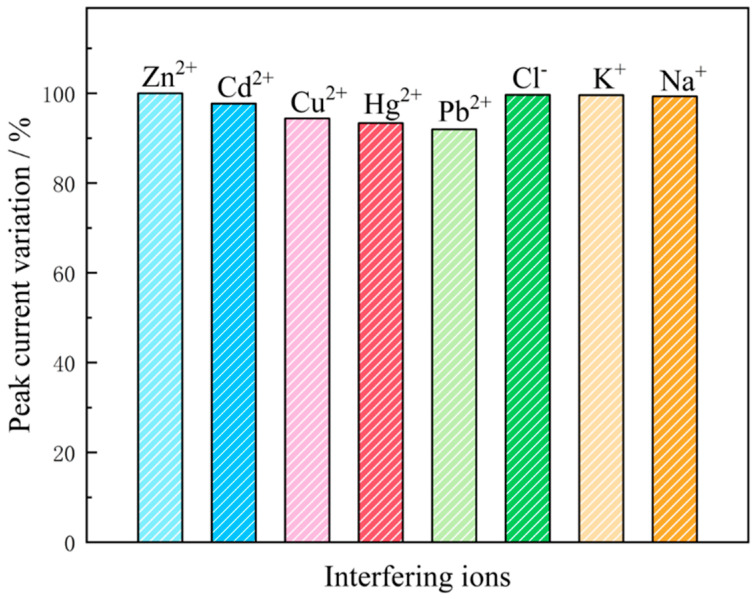
Differences in peak currents after addition of 50−fold Cl^−^, K^+^, Na^+^, Cd^2+^, Cu^2+^, Hg^2+^ and Pb^2+^ ions to a buffer solution containing 300 μg/L of zinc ions.

The anti-interference experiments conducted in a buffer solution containing 300 μg/L of Zn(II) revealed that the changes in the peak currents for the measured values of Zn(II) are all less than 10% for 50 times the concentrations of Cl^−^, K^+^ and Na^+^ and 10 times the concentrations of Cd^2+^, Cu^2+^, Hg^2+^ and Pb^2+^. This demonstrates that the anti-interference capabilities of the improved electrode for Zn(II) detection are superior (Figure 14).

### 3.6. Sensitivity Analysis

The detection impacts of various electrodes on zinc ions in recent years were examined to better investigate the detection performance of the microfluidic system developed in this study, as shown in Table 3. The detection range and detection limit of the GR/CeO_2_/Nafion/Au microfluidic system developed in this study vary slightly compared with the corresponding results of other electrodes reported in previous studies, with a wider detection range and lower detection limit, indicating that the developed system can be used for the detection of zinc ions in seawater. This is supported by the comparison results with those of other studies.

### 3.7. Real Sample Detection

To assess the practicability of the developed system, we collected near-shore seawater from the Maowei Sea and Qinzhou Harbour (both are semi-enclosed bays located in southern China) for spiking and recovery experiments. Sample 1 consists of seawater from the Maowei Sea mouth, while Sample 2 is near-shore seawater obtained from Qinzhou Harbour. For complete precipitation of the contaminants, the samples were left to stand for 3 days. After filtering the supernatant of the water samples with a 0.45 μm filter membrane, the seawater samples were combined with the HAc–NaAc solution at a 1:1 ratio. The SWV method was then used to ascertain the results under ideal conditions. Table 4 shows that the results of the microfluidic system developed in this study are close to the detection results of the inductively coupled plasma-mass spectrometry (ICP-MS) method, and the average recoveries of the spiked recovery experiments ranged from 99.2% to 100.0%, indicating that the GR/CeO_2_/Nafion/Au sensor is highly accurate and can be used for the rapid and precise detection of Zn(II) in seawater and other water bodies.

## 4. Conclusions

In this study, a graphene/CeO_2_/Nafion-modified planar disc electrode was used to detect Zn(II) in seawater using SWV and the optimal conditions were determined using orthogonal tests. Under the optimal conditions, the determination of Zn(II) in the range of 1–900 μg/L was achieved, with a limit of detection of 0.87 μg/L. GR/CeO_2_/Nafion/Au exhibited good stability, reproducibility and anti-interference. The system was used for the determination of Zn(II) in seawater, and the results were highly similar to those of the ICP-MS method. The spiked recoveries ranged from 99.2% to 100.0%, which proved that the sensor could be used for highly sensitive and accurate detection of Zn(II) in seawater.

## Figures and Tables

**Table 1:** Orthogonal test protocols and analysis of the results.

Experiment	GR (mg·mL^−1^)	CeO_2_ (mg·mL^−1^)	Nafion (μL)	Film Thickness (μL)	Peak Current (μA)
1	1	0.1	3	5	15.75
2	1	0.2	4	6	17.02
3	1	0.3	5	7	9.32
4	1.5	0.1	4	7	18.57
5	1.5	0.2	5	5	22.06
6	1.5	0.3	3	6	21.93
7	2	0.1	5	6	17.83
8	2	0.2	3	7	14.03
9	2	0.3	4	5	16.26

**Table 2:** Visual analysis table of orthogonal tests.

Average	GR (mg·mL^−1^)	Peak Current (μA)	CeO_2_ (mg·mL^−1^)	Peak Current (μA)	Nafion (μL)	Peak Current (μA)	Film Thickness (μL)	Peak Current (μA)
I	1	14.030	0.1	17.383	3	17.237	5	18.023
II	1.5	20.853	0.2	17.703	4	17.283	6	18.927
III	2	16.040	0.3	15.837	5	16.403	7	13.973
Range *R*	–	6.823	–	1.866	–	0.880	–	4.954

**Table 3:** Comparison of different electrodes.

Electrodes	Linear Range (μg·L^−1^)	LOD (μg·L^−1^)	Reference
Bi/GO/GCE	20–8000	6	[23]
Hg/CMWCNTs@ZIF-8/GCE	50–1000	5.23	[24]
Calix/MPA/Au	2850–6650	1500	[25]
Ti_3_C_2_(HF)/Fe_3_O_4_/gC_3_N_4_	0.325–32.5	0.0169	[26]
GR/CeO_2_/Nafion/GCE	5–800	1.20	[27]
GR/CeO_2_/Nafion/Au	1–900	0.87	This work

**Table 4:** Detection results of zinc ion concentration in actual samples.

Seawater Samples	Found (μg·L^−1^)	ICP-MS	Added (μg·L^−1^)	Found (μg·L^−1^)	Recovery (%)	RSD (%) (*n* = 3)
1	16.04 ± 0.39	16.0	0	—	—	—
			10	26.02 ± 0.34	99.9	1.37
			20	35.76 ± 0.37	99.2	1.05
2	546.23 ± 0.72	546.0	0	—	—	—
			10	554.15 ± 0.78	99.6	0.14
			20	566.21 ± 0.91	100.0	0.16

## Data Availability

Data are contained within the article.
